# Anti-inflammatory treatment after cataract surgery in Sweden: changes in prescribing patterns from 2010 to 2017

**DOI:** 10.1136/bmjophth-2020-000635

**Published:** 2021-03-23

**Authors:** Behrad Samadi, Mats Lundstrom, Madeleine Zetterberg, Ingela Nilsson, Per Montan, Anders Behndig, Maria Kugelberg

**Affiliations:** 1 Department of Clinical Neuroscience, Karolinska Institute, Stockholm, Sweden; 2 Department of Clinical Sciences, Ophthalmology, Faculty of Medicine, Lund University, Karlskrona, Sweden; 3 Department of Clinical Neuroscience, University of Gothenburg, Mölndal, Sweden; 4 Ophthalmology, Sahlgrenska University Hospital, Goteborg, Sweden; 5 Capio Medocular AB, Malmö, Sweden; 6 Department of Clinical Neuroscience, Karolinska Institute, St Erik Eye Hospital, Stockholm, Sweden; 7 RC Syd, Blekinge Hospital Karlskrona, Karlskrona, Sweden

**Keywords:** treatment surgery, inflammation, treatment medical

## Abstract

**Aims:**

To investigate changes in the prescribing patterns of postoperative eye drops following cataract surgery in Sweden from 2010 to 2017.

**Methods:**

Data from cataract procedures registered in the National Cataract Register during the month of March from 2010 to 2017 were record linked and sent to the Swedish Prescribed Drug Register, which allowed us to determine which eye drops the patients had obtained from 3 months presurgery to 2 weeks post surgery.

**Results:**

During the 8-year study period, 54 889 surgeries were registered. Combination treatment with steroid and non-steroidal anti-inflammatory drug (NSAID) eye drops increased from 12% in 2010 to 60% in 2017 (p<0.001) while monotherapy with steroids decreased from 71% in 2010 to 26% in 2017 (p<0.001). Monotherapy with NSAIDs after surgery was fairly stable, at 17% in 2010 and 13% in 2017 (p<0.001). Combination treatment was more frequent in patients with diabetic retinopathy (p<0.001) or age-related macular degeneration (p<0.001), while monotherapy with steroids was more frequent in patients with glaucoma (p<0.001). The proportion of monotherapy or combination therapy varied widely between ophthalmic clinics. The prescription of antibiotic eye drops after surgery also varied greatly between clinics, from 0% to 63%, with a national average of 4.9%.

**Conclusion:**

There is a change in the prescription pattern of anti-inflammatory eye drops after cataract surgery in Sweden, with less monotherapy and an increasing proportion of patients receiving a combination of steroid and NSAID eye drops.

**Key messages**

**What is already known about this subject?**

- Non-steroidal anti-inflammatory drugs (NSAIDs), and steroid eye drops are regularly prescribed after cataract surgery; however, limited data currently exist regarding the prescribing patterns of these eye drops in Sweden.
- Since the introduction of intracameral antibiotics during cataract surgery, the prescription of postoperative topical antibiotics has diminished in Sweden. However, in the USA in 2016, antibiotic eye drops were the most commonly prescribed drug class per volume.

**What are the new findings?**

- From 2010 to 2017, there was a remarkable change in the prescribing patterns of postoperative eye drops following cataract surgery in Sweden.
- Combination treatment with steroid and NSAID eye drops increased greatly, while steroid eye drops as monotherapy decreased substantially.
- The prescription of postoperative antibiotic eye drops remained low.

**How might these results change the focus of research or clinical practice?**

- In Sweden, there might be a redundancy in postoperative treatment following cataract surgery in eyes with low risk of developing pseudophakic macular oedema. As such NSAIDs, particularly as a combination treatment with steroid eye drops, should perhaps be reserved for high-risk cases where it is warranted.
- Postoperative topical antibiotics, as endophthalmitis prophylaxis, might not be a necessity when intracameral antibiotics have already been given during cataract surgery.
- For future studies, looking into the variability of costs related to the different eye drops prescribed for cataract surgery, may improve treatment protocols and management of healthcare expenditures.

## Introduction

Cataract surgery is the most commonly performed surgical procedure worldwide. Its primary purpose is to improve patients’ visual function. The procedure causes a disruption of the blood-aqueous barrier and intraocularly releases crystalline lens particles, which induces a postoperative inflammation.^1–3^ If inadequately treated, the inflammation can lead to further vision-disturbing complications, such as posterior synechia, secondary glaucoma, posterior capsular opacification and pseudophakic macular oedema (PMO),^4–7^ lowering the quality of life for the patients and generating substantially higher costs for the healthcare system.^8 9^ Postoperative inflammation after cataract surgery is regularly treated according to local clinical routines and traditions but the treatment can also be surgeon dependent. Some patients are treated with topical steroids, some with non-steroidal anti-inflammatory drug (NSAID) eye drops and others with a combination of both. In the last decade, NSAID eye drops, which potentially have fewer side effects but cost much more than steroidal eye drops, have become increasingly popular after clinical trials have shown their inhibiting effect on postsurgical macular swelling and implied a potential to reduce the incidence of PMO.^9 10^ However, the incidence of PMO varies widely between studies,^7 11 12^ and despite many recent reports, there is no convincing evidence as to which treatment has the highest overall therapeutic effect or the best cost-benefit profile.

In view of the lack of compelling evidence of the superiority of a specific treatment strategy, we considered it important to conduct this register-based study, through data coupled to the Swedish Prescribed Drug Register (SPDR), to gain knowledge of changes in the prescribing patterns of anti-inflammatory treatment following cataract surgery in Sweden.

## Materials and methods

All ophthalmic clinics in Sweden report baseline data to the Swedish National Cataract Register (NCR), covering more than 96% of all cataract procedures performed nationwide, which in 2018 corresponded to 133 973 cataract surgeries.^13^ Baseline registration includes, among other data, social security number for identification, laterality, date of surgery and ocular comorbidity (glaucoma, diabetic retinopathy (DRP), age-related macular degeneration (AMD), corneal guttata or ‘other sight-threatening ocular comorbidity’). The study began in early 2018 with retrospective data collection. To limit the extensive amount of data from the register needed for our study, we chose to collect data from the month of March each year from 2010 through 2017. March is a representative month during which approximately one tenth of the annual cataract surgeries are performed. During these 8 years, 54 889 surgeries were performed, during the months of March, which were included in the study and subsequently sent to the SPDR held by the National Board of Health and Welfare. To ensure that the dispensed prescriptions covered the surgical time frame, we requested prescriptions that had been made between 3 months prior to the surgery, and 2 weeks after the date of the surgery. This is because some clinics routinely prescribe eye drops preoperatively while others prescribe them at the time of surgery. Sometimes, when the clinic forgets to prescribe eye drops, medications are prescribed a few days post surgery. The following Anatomical Therapeutic Chemical Classification codes were searched for: S01BA01, S01BC03, S01BC10, S01BC11, S01CA01, S01AE07, S01AA01, S01AA12 and S01AA13. These codes cover steroids, NSAIDs and antibiotic eye drops licensed in Sweden. Eventually, we retrieved deidentified data from the SPDR on the drugs collected by the patients during the specified time frame.

Comorbidities such as DRP, AMD and glaucoma are registered in the NCR. We specifically investigated prescription patterns of postoperative eye drops for patients with these diagnoses assuming that the treatment regimens would be different from those offered to patients not suffering from sight-threatening ocular conditions. All cataract surgeries were included.

Furthermore, we wanted to determine whether prescription patterns differed between ophthalmology departments. Therefore, we chose to investigate the distribution of postoperative anti-inflammatory eye drops in departments that performed more than a total of 1000 cataract procedures during the studied years.

In Sweden, practically all patients are given intracameral antibiotics at the end of their cataract procedure,^13^ which might obviate the need for postoperative topical antibiotic treatment after cataract surgery as shown in the European Society of Cataract & Refractive Surgeons endophthalmitis study from 2007.^14^ Yet some surgeons prescribe them. Hence, we also wanted to investigate the extent of this practice.

### Statistical analyses

The χ^2^ test, using statistical software R V.3.6.1, was used to analyse statistical differences between and within groups. A p value less than 0.05 was considered as statistically significant.

### Patient involvement

Patients were not directly involved in the design of this study.

## Results

**Figure 1:**
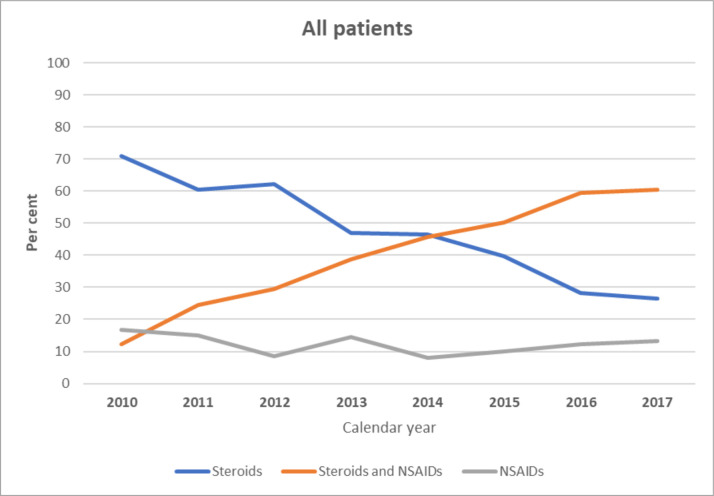
Overall change in dispensed anti-inflammatory eye drops after cataract surgery from 2010 to 2017. NSAID, non-steroidal anti-inflammatory drug.

**Figure 2:**
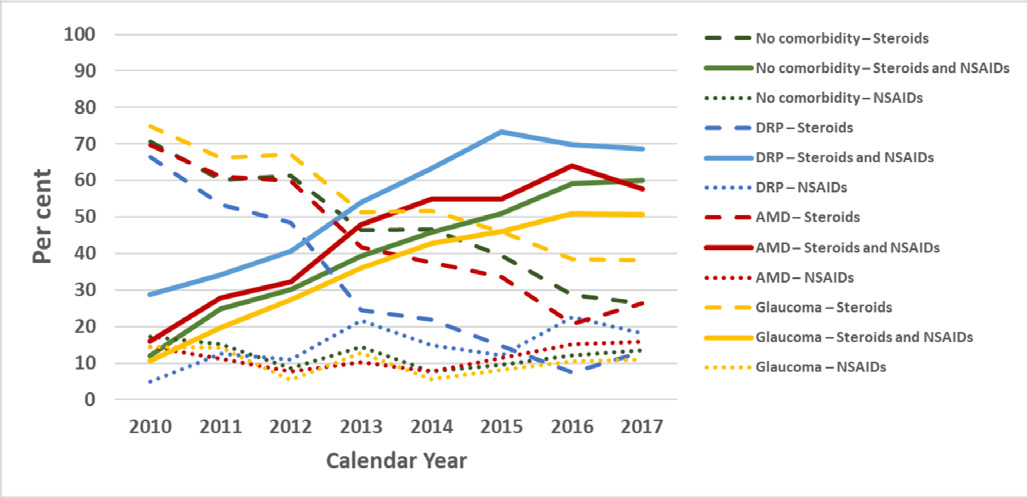
Changes in dispensed anti-inflammatory eye drops after cataract surgery in patients with and without ocular comorbidities, from 2010 to 2017. AMD, age-related macular degeneration; DRP, diabetic retinopathy; NSAID, non-steroidal anti-inflammatory drug.

Among the 54 889 surgeries, a dispensed prescription for postoperative treatment (steroids or NSAIDs, or both, and/or antibiotic eye drops) was found in 45 559 (83%) and for steroids and/or NSAIDS in 43 296 (79%). As figure 1 shows, the pattern of dispensed eye drops changed dramatically over the years studied. Overall, prescriptions for a combination of steroids and NSAIDs increased from 12% during 2010 to 60% in 2017 (p<0.001), while steroid monotherapy dropped from 71% to 26% (p<0.001). Monotherapy with NSAIDs decreased slightly during the years, from 17% to 13% (p<0.001). Figure 2 shows the change in the prescription pattern in different subgroups including no comorbidity and each of the ocular comorbidities. Moreover, the number of patients to whom anti-inflammatory eye drops were dispensed, as registered in the SPDR each year from 2010 to 2017, can be seen in the online supplemental table.

10.1136/bmjophth-2020-000635.supp1 Supplementary data

In total, over the entire 8-year study period, patients with DRP received combination treatment to a greater extent than patients without DRP (p<0.001). Combination treatment was also prescribed more often to patients with AMD than to patients without AMD (p<0.001). In patients with glaucoma, monotherapy with steroids was more common than in patients without glaucoma (p<0.001), table 1.

**Table 1:** Total number of patients to whom anti-inflammatory eye drops were dispensed after cataract surgery, by ocular comorbidity, 2010–2017

	DRP				P value
	No		Yes		
	N	% of No	N	% of Yes	
DRP					
Steroids	19 803	47.8	559	30.1	<0.001*
Steroids and NSAIDs	16 619	40.1	1020	55	
NSAIDs	5018	12.1	277	14.9	
AMD					
Steroids	17 189	47.6	3173	44.1	<0.001†
Steroids and NSAIDs	14 464	40.1	3175	44.2	
NSAIDs	4453	12.3	842	11.7	
Glaucoma					
Steroids	18 270	46.3	2092	54.1	<0.001*
Steroids and NSAIDs	16 259	41.2	1380	35.7	
NSAIDs	4903	12.4	392	10.1	

Note that the table includes all the patients under each of the three subgroups diabetic retinopathy (DRP), age-related macular degeneration (AMD), and glaucoma. Thus, for example, a patient listed under ‘Yes’ for DRP might also be listed under ‘Yes’ for AMD, and a patient under ‘Yes’ for DRP might also be listed under ‘No’ for glaucoma.

*In pairwise comparisons using χ^2^ tests, all combinations were statistically significant.

†In pairwise comparisons using χ^2^ tests, all combinations were statistically significant except for steroids versus steroids and non-steroidal anti-inflammatory drugs (NSAIDs) (p-value 0.98).

**Figure 3:**
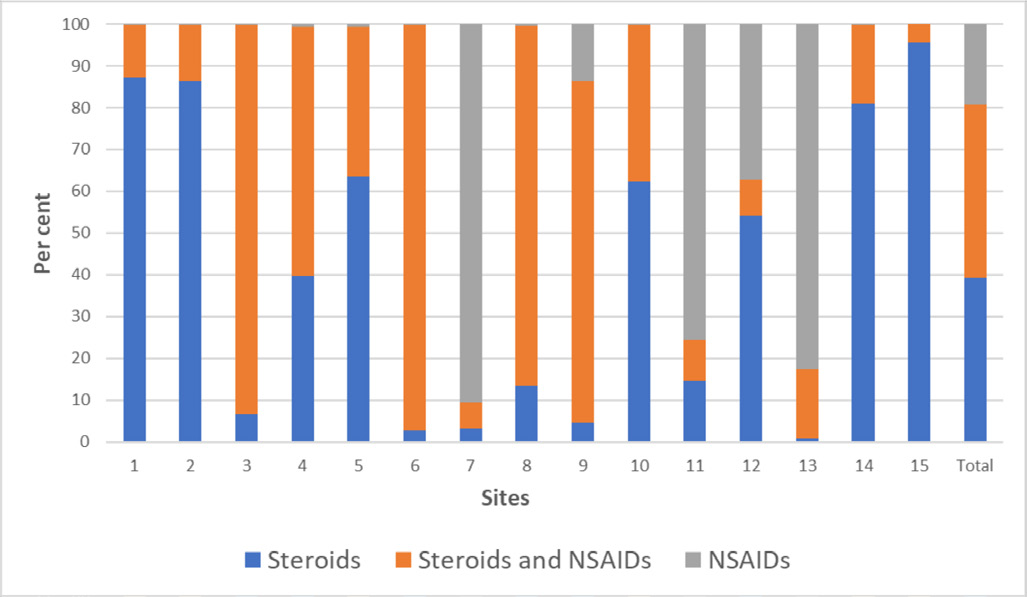
Distribution, per clinic (site), of anti-inflammatory eye drops dispensed after cataract surgery. NSAID, non-steroidal anti-inflammatory drug.

There was a large difference in prescription patterns between clinics, as can be seen in figure 3. The prescription of antibiotic eye drops, either alone or in combination with steroids and/or NSAIDs, differed widely between clinics, ranging from 0% to 63%. The national average for topical antibiotic prescription was 4.9% following cataract surgery.

## Discussion

In the current register-based study, we analysed prescription patterns of postoperative eye drops in patients who underwent cataract surgery in Sweden from 2010 to 2017. The results showed that postoperative eye drops with either steroids or NSAIDs or both were dispensed in 79% of cataract surgeries, signifying that 21% did not retrieve any anti-inflammatory eye drops after surgery. This might be explained by clinics giving subconjunctival steroids at the end of surgery instead of pursuing the more common path of topical postoperative treatment, and some patients who are unable to collect eye drops from the pharmacy obtaining them from the clinic before they are discharged. Also, some patients have an unused bottle dispensed from the first eye surgery, which explains why some patients did not collect the prescription made for the second eye. These patients are not recorded in the SPDR.

In a recently published article by Zafar *et al*,^15^ it was reported that, out of 591 733 American patients who underwent cataract surgery in 2016, 88% had at least one eye drop prescription in the postoperative period, which included monotherapy or any combination of steroids, NSAIDs or antibiotics. Similar to our study, the authors concluded that the 12% who did not receive prescriptions probably received periocular treatment during surgery or obtained eye drops before the dates covered by their analysis.

Another interesting comparison between our studies concerns postoperative antibiotics, which showed a striking difference. While in our study the least common medication prescribed after cataract surgery in Sweden was antibiotic eye drops (4.9%), Zafar *et al*^15^ by contrast concluded that they are the most common by volume (89%) in the USA, generating enormous costs. Nevertheless, we found individual clinics prescribing antibiotic eye drops in up to 63% of their cataract surgeries. In the current study, only 13 out of the 54 889 eyes operated (0.0002%) did not receive any intracameral antibiotics. Since the introduction of intracameral antibiotics, the endophthalmitis rate after cataract surgeries in Sweden has decreased dramatically from 0.1% in 1998 to 0.012% in 2019.^16^ In a Swedish study from 2013, the 6-year incidence of endophthalmitis after cataract surgery from 2005 through 2010 was found to be 0.029%.^17^ Considering that intracameral antibiotics are given to practically all patients in Sweden during cataract surgery, we believe that prescribing additional antibiotic eye drops postoperatively might not further prevent endophthalmitis, as previous studies have shown,^14 18^ but be less cost effective,^19^ and might even compromise the compliance to the anti-inflammatory regimen by imposing the extra burden of having to take additional drops, which is a recognised issue.^20 21^

In our study, steroid eye drops, either as monotherapy or as part of a combination therapy, represented the highest prescribed postoperative medication by volume (87.7%), followed by NSAID eye drops (52.9%). In the study by Zafar *et al*,^15^ steroids represented the second highest medication group by prescription volume (86%), while NSAIDs were the third highest (66%). Analysing the prescription pattern from 2010 to 2017, our study showed that steroid monotherapy overall decreased from 71% to 26%, while combination of steroids and NSAIDs increased fivefold from 12% during 2010 to 60%. in 2017. The overall trend in prescribing NSAID eye drops as monotherapy remained fairly stable throughout the study period, although an increase was seen in patients with DRP undergoing cataract surgery.

Likewise, a closer look at each of the three ocular comorbidity groups and the no comorbidity group, showed that there was a significant upward trend in combination prescriptions and a decline in monotherapy with steroids. In the no comorbidity group alone, combination treatment increased by almost six times, as shown in figure 2. Over the study period, postoperative combination treatment was most commonly prescribed in the DRP group (55%) and the AMD group (44%), while steroid monotherapy remained the highest prescribed postoperative treatment in the glaucoma group (54%). Despite the well-known increased risk of steroid response in glaucoma patients, one explanation for the outcome in this group may be that the majority of these patients are already on one or several antihypertensive eye drops; therefore, adding several drops could affect compliance, which has already been shown to be an issue.^20 21^ Also, many glaucoma patients already have ocular surface issues due to their glaucoma drops. While steroid eye drops might temporarily relieve some of these issues, one should try to avoid the additional local adverse effects that NSAID drops may have. Some NSAID drops have been associated with keratopathy, corneal melts and severe allergic reactions,^10 22^ and in an article by Ylinen *et al*,^23^ ocular symptoms were reported to be more frequent in patients taking NSAID drops alone or in combination than in patients on corticosteroids alone.

Nevertheless, the overall prescription rate of NSAID eye drops after cataract surgery has increased over the years as a growing number of articles, as outlined below, have addressed the efficacy of these drops in controlling postoperative inflammation and decreasing the incidence of PMO. However, the incidence of PMO varies a lot in different studies, depending on the definition of ‘PMO’, and on which instrument has been used to measure the oedema, and whether it is affecting the visual function. Studies using fluorescein angiography have reported incidences of 9%–19%,^7 11^ while Lobo *et al*^12^ using optical coherence tomography showed an incidence of PMO, 6 weeks postoperatively, of up to 41%. However, in studies correlating PMO with a decline in postoperative visual acuity the incidence ranged from 0.6% to 4%,^12 24–26^ with a higher incidence in patients with diabetes and uveitis.^6 27 28^ In fact, several studies have shown that diabetes is a risk factor for developing PMO^6 29 30^ and that diabetic patients receiving combination treatment have a significantly lower incidence of PMO compared with those receiving steroid eye drops alone.^31^ In 2017, the London-based Royal College of Ophthalmologists adopted the National Institute for Health and Care Excellence guidelines on the management of cataracts in adults, in which it is stated that the surgeon should consider postoperative combination treatment for patients at increased risk of PMO, such as patients with diabetes or uveitis.^32^ This might explain the higher NSAID prescription rate among the DRP patients in our study.

Nevertheless, two separate review articles concluded that although topical NSAIDs reduced the incidence of PMO after cataract surgery, they did not have a clinically relevant effect on mean visual acuity.^33 34^ It might be argued that the main aim of cataract surgery is to improve visual function, in which case the additional cost of NSAID eye drops would be unjustified since they only reduce the small risk of PMO causing an anatomic (rather than a functional) complication. Moreover, in a systematic review Kim *et al*^10^ argue that in most healthy eyes, PMO resolves spontaneously, without altering the long-term (>3 months) visual outcome, questioning the need to prescribe NSAID drops.

In a randomised trial, Ylinen *et al*^23^ concluded that combination therapy following cataract surgery was superior to stand-alone treatment with either steroid or NSAID drops in reducing the incidence of PMO. However, the follow-up was at 1 month, whereas the peak incidence of PMO usually occurs beyond 5 weeks.^35^ The recently published Prevention of Macular Edema study showed that the incidence of clinically significant PMO after cataract surgery in otherwise healthy eyes within 12 weeks postoperatively was lowest in the group receiving combination therapy, followed by the group receiving stand-alone NSAID treatment, with the steroid group having the highest incidence. However, the results were not statistically significant after Bonferroni correction.^36^

Hence, despite much research published in this area, there is no convincing evidence as to which anti-inflammatory treatment regimen is superior or more cost effective after cataract surgery, which reflects the treatment variability between clinics as outlined in figure 3. As a stand-alone treatment, NSAID eye drops, comprising almost 80% of prescriptions in two of the busiest cataract clinics (>1000 cataract surgeries per year) in Sweden, might not be warranted considering the higher cost and lack of consistent evidence of effectiveness compared with steroid eye drops. However, given current evidence as outlined above, combination treatment for prevention of PMO, which was most commonly prescribed in five of the busiest clinics, should perhaps be reserved for patients with DRP or other high-risk ocular comorbidities, as recommended by the 2016 American Academy of Ophthalmology Preferred Practice Pattern guidelines.^37^ Needless to say, more research is needed to investigate the long-term benefits of the various anti-inflammatory eye drops following cataract surgery.

Our study had some important limitations which could partly be explained due to our anonymised data set. However, we do not believe that these limitations changed the overall conclusion of the study. Unfortunately, it was not possible to obtain information on prescribed eye drops that were not dispensed as these data are stored for only a few months in Sweden. Given that NSAID eye drops are more expensive than steroid eye drops, this valuable information would have allowed us to establish whether patients chose not to collect their medication because of cost issues. Moreover, to limit the vast amount of data, we only looked at cataract surgeries performed during the months of March. Perhaps looking at the data sets over the entire years might have affected the outcome. Furthermore, we did not compare the patient demographics between clinics, such as differences in the number of patients with ocular co-morbidities. However, as we compared clinics that performed over 1000 cataract surgeries annually, we assumed the distribution to be quite similar. Also, we did not investigate the specifics to why 21% were not dispensed eye drops postoperatively in our data set; however, as mentioned previously we have an understanding that there are clinics with alternate treatment protocols and retrieval of eye drops for patients. Finally, when comparing patients with and without each of the three subgroups of comorbidities (DRP, AMD, glaucoma), we did not exclude other comorbidities under each category. Thus, a patient with AMD might have also had glaucoma, which could have affected the treatment protocol. Likewise, in cataract surgeries involving complications the treatment protocol might have differed compared with routine cataract surgeries. These cases were not analysed separately.

In summary we found that there was a clear change in the prescription pattern of anti-inflammatory eye drops after cataract surgery in Sweden from 2010 to 2017. Overall, prescriptions combining steroid and NSAID eye drops substantially increased while traditional monotherapy with steroid eye drops decreased considerably. The prescription of antibiotic eye drops remained low. Changes in prescribing patterns can generate huge costs for the healthcare system and for the patients. In future studies, it would be of great interest to look further into the variability of costs related to the different eye drops prescribed to optimise treatment and help control expenditures.

## Data Availability

All data relevant to the study are included in the article or uploaded as supplementary information.
