# Supplementary material for: Anti-inflammatory treatment after cataract surgery in Sweden: changes in prescribing patterns from 2010 to 2017
Source: BMJ Open Ophthalmol. 2021 Mar 23;6(1):e000635. doi: 10.1136/bmjophth-2020-000635 (PMC7993195; doi:10.1136/bmjophth-2020-000635)
Supplement: Supplementary data [file bmjophth-2020-000635supp001.pdf]

Number and percentage of patients to whom anti-inflammatory eye drops were dispensed following cataract surgery from 2010 through 2017

ATC combinations	2010		2011		2012		2013		2014		2015		2016		2017		Total	
All patients	**n**	**%**	**n**	**%**	**n**	**%**	**n**	**%**	**n**	**%**	**n**	**%**	**n**	**%**	**n**	**%**	**n**	**%**
Steroids	3,724	71.0	3,148	60.3	2,899	62.2	2,471	46.9	2,895	46.5	2,161	39.7	1,471	28.3	1,593	26.4	20,362	47.0
Steroids and NSAIDs	643	12.3	1,281	24.6	1,371	29.4	2,036	38.7	2,843	45.6	2,734	50.2	3,093	59.5	3,638	60.3	17,639	40.7
NSAIDs	876	16.7	788	15.1	393	8.4	760	14.4	493	7.9	547	10.1	638	12.3	800	13.3	5,295	12.2
**Total**	**5,243**	**100**	**5,217**	**100**	**4,663**	**100**	**5,267**	**100**	**6,231**	**100**	**5,442**	**100**	**5,202**	**100**	**6,031**	**100**	**43,296**	**100**
Patients with DRP																		
Steroids	138	66.3	106	53.3	111	48.5	59	24.5	65	22.0	33	14.7	17	7.4	30	13.0	559	30.1
Steroids and NSAIDs	60	28.8	68	34.2	93	40.6	130	53.9	187	63.2	164	73.2	160	69.9	158	68.7	1,020	55.0
NSAIDs	10	4.8	25	12.6	25	10.9	52	21.6	44	14.9	27	12.1	52	22.7	42	18.3	277	14.9
**Total**	**208**	**100**	**199**	**100**	**229**	**100**	**241**	**100**	**296**	**100**	**224**	**100**	**229**	**100**	**230**	**100**	**1,856**	**100**
Patients with AMD																		
Steroids	679	69.6	542	61.0	498	60.0	367	41.8	372	37.6	301	33.6	165	20.8	249	26.4	3,173	44.1
Steroids and NSAIDs	155	15.9	248	27.9	268	32.3	421	47.9	542	54.8	491	54.9	507	64.0	543	57.6	3,175	44.2
NSAIDs	141	14.5	99	11.1	64	7.7	90	10.3	75	7.6	103	11.5	120	15.2	150	15.9	842	11.7
**Total**	**975**	**100**	**889**	**100**	**830**	**100**	**878**	**100**	**989**	**100**	**895**	**100**	**792**	**100**	**942**	**100**	**7190**	**100**
Patients with glaucoma																		
Steroids	371	74.8	296	66.2	314	67.2	238	51.3	296	51.7	203	45.8	176	38.5	198	38.2	2,092	54.1
Steroids and NSAIDs	53	10.7	88	19.7	128	27.4	167	36.0	244	42.7	204	46.0	233	51.9	263	50.8	1,380	35.7
NSAIDs	72	14.5	63	14.1	25	5.4	59	12.7	32	5.6	36	8.1	48	10.5	57	11.0	392	10.1
**Total**	**496**	**100**	**447**	**100**	**467**	**100**	**464**	**100**	**572**	**100**	**443**	**100**	**457**	**100**	**518**	**100**	**3,864**	**100**

AMD = age-related macular degeneration; ATC = Anatomical Therapeutic Chemical Classification code; DRP = diabetic retinopathy; NSAIDs = non-steroidal anti-inflammatory drugs.
